# Detection of residual rifampicin in urine via fluorescence quenching of gold nanoclusters on paper

**DOI:** 10.1186/s12951-015-0105-5

**Published:** 2015-06-26

**Authors:** Krishnendu Chatterjee, Chiung Wen Kuo, Ann Chen, Peilin Chen

**Affiliations:** Department of Engineering and System Science, National Tsing Hua University, Hsinchu, 300 Taiwan; Nano Science and Technology Program, Taiwan International Graduate Program, Academia Sinica, Taipei, 115 Taiwan; National Tsing Hua University, Hsinchu, 300 Taiwan; Research Center for Applied Sciences, Academia Sinica, Taipei, 115 Taiwan, ROC; Department of Pathology, Tri-Service General Hospital, National Defense Medical Center, Taipei, Taiwan

**Keywords:** Fluorescent biosensors, Fluorescence quenching, Gold nanocluster, Rifampicin detection, TB drug monitoring, Wax-printed paper platform

## Abstract

**Background:**

Rifampicin or rifampin (R) is a common drug used to treat inactive meningitis, cholestatic pruritus and tuberculosis (TB), and it is generally prescribed for long-term administration under regulated dosages. Constant monitoring of rifampicin is important for controlling the side effects and preventing overdose caused by chronic medication. In this study, we present an easy to use, effective and less costly method for detecting residual rifampicin in urine samples using protein (bovine serum albumin, BSA)-stabilized gold nanoclusters (BSA-Au NCs) adsorbed on a paper substrate in which the concentration of rifampicin in urine can be detected via fluorescence quenching. The intensity of the colorimetric assay performed on the paper-based platforms can be easily captured using a digital camera and subsequently analyzed.

**Results:**

The decreased fluorescence intensity of BSA-Au NCs in the presence of rifampicin allows for the sensitive detection of rifampicin in a range from 0.5 to 823 µg/mL. The detection limit for rifampicin was measured as 70 ng/mL. The BSA-Au NCs were immobilized on a wax-printed paper-based platform and used to conduct real-time monitoring of rifampicin in urine.

**Conclusion:**

We have developed a robust, cost-effective, and portable point-of-care medical diagnostic platform for the detection of rifampicin in urine based on the ability of rifampicin to quench the fluorescence of immobilized BSA-Au NCs on wax-printed papers. The paper-based assay can be further used for the detection of other specific analytes via surface modification of the BSA in BSA-Au NCs and offers a useful tool for monitoring other diseases.

**Electronic supplementary material:**

The online version of this article (doi:10.1186/s12951-015-0105-5) contains supplementary material, which is available to authorized users.

## Background

Rifampicin is an important bactericidal antibiotic drug of the rifamycin group, which inhibits DNA-dependent RNA polymerase and suspends bacterial growth. This drug is widely used to target *Bacillus* and *Listeria* strains and commonly prescribed together with other drugs for the treatment of tuberculosis (TB) [1–3] and infections associated with osteomyelitis, prosthetic joints, and meningitis. In the treatment of TB, three frontline drugs, isoniazid (INZ), ethambutol (E) and pyrazinamide (P), are given together with rifampicin. In a standard treatment procedure for TB, all four drugs are administered in various combinations over the first 2 months, and isoniazid and rifampicin are continued for the next 4 months [1]. Improper dosage during the sustained medication period (2–6 months) often leads to drug resistance and even death despite the diseases being curable. Therefore, it is necessary to monitor possible irregularities in drug dosage. The simplest approach for detecting irregularity in drug dosage is to assess the residual rifampicin in the urine of patients; however, inaccurate results could adversely affect the patient and lead to an increased risk of hepatotoxicity because of increased ingestion and drug accumulation in the liver. Thus, various methods have been applied to detect rifampicin levels, including spectrophotometry [4], chemiluminescence [5], electrochemistry [6], and high-performance liquid chromatography (HPLC) [7–11]. However, it is desirable to develop an alternative approach for on-site detection of residual rifampicin in urine. Recently, a paper-based sensing platform was demonstrated as an attractive alternative to conventional analytical instrumentation for point-of-care medical diagnostics [12, 13], and it is ideal for the detection of residual rifampicin in urine because of its advantages, including its low cost, quick response time, ease of handling and disposability. However, the selectivity and sensitivity of paper-based sensors are highly dependent on the sensing scheme.

With recent developments in nanotechnology, nanoparticle-based sensors have demonstrated notably high sensitivity in various applications [14–19]. Fluorescent metal nanoclusters (NCs) [20–27] are a recent addition to a growing list of labeling and sensing agents that have generated massive interest among researchers because of their biocompatibility, non-toxicity and outstanding fluorescent properties. Fluorescent gold nanoclusters manufactured via eco-friendly one-pot synthesis approaches using commonly available templating agents (i.e., bovine serum albumin (BSA) [20], lysozyme [21], rec1-resilin [22], transferrin [23], glutathione [24], insulin fibrils [25], horseradish peroxidase [26] and papain [27]) have been reported by several groups. Among these materials, BSA-stabilized metal nanoclusters have been widely used as fluorescent turn-off probes for the detection of methotrexate (MTX) [28], quercetin [29], cyanide [30], glucose [31], Hg^2+^ [32], and Cu^2+^ in aqueous solution [33] and even Cu^2+^ in live cells [34]. To date, various NC applications have been reported as fluorescent probes used in the solution phase [28–34]. However, few studies [35] have explored the immobilization of NCs on paper and their subsequent use as a sensing platform. Based on the concept of paper chromatography, immobilized paper sensors have been used in modern laboratories for a number of years. However, applications with paper-based drug monitoring devices have not been fully explored.

**Figure 1:**
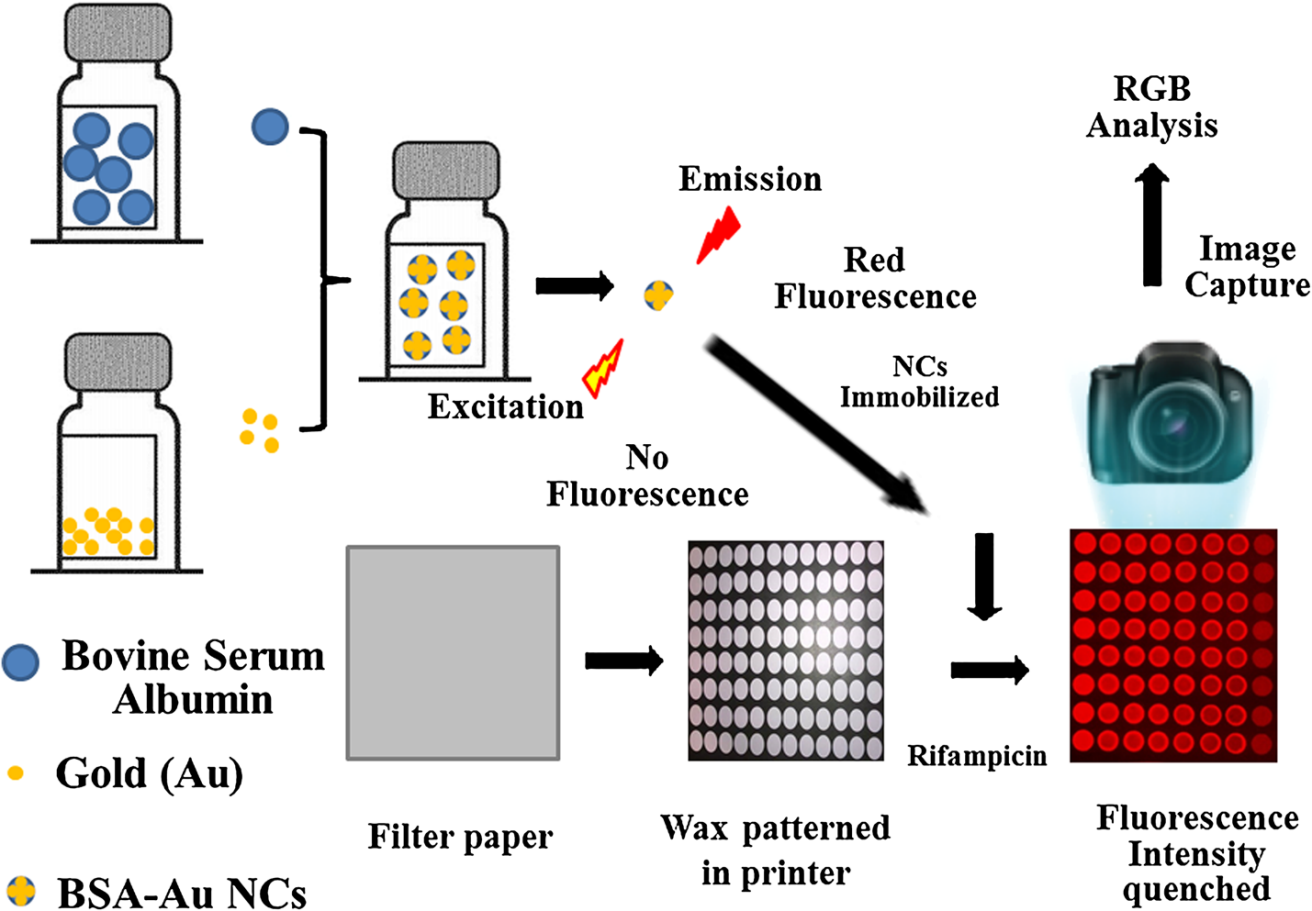
Schematic illustration of the synthesis, BSA-Au NC immobilization on paper and application to detect rifampicin.

In this experiment, a commercially available printer and hot plate were used to create multi-zone standard 96-well paper microplates as an alternative to conventional polymer multiwell plates [12, 13]. The fluorescent BSA-Au NCs were immobilized on a wax-printed paper platform in which the fluorescence intensity from the immobilized BSA-Au NC was quenched in the presence of rifampicin. Rifampicin exhibited a greater binding affinity with BSA than isoniazid, and the binding order of rifampicin with BSA is approximately 10^4^ L/mol [36], whereas it is approximately 10^3^ L/mol [37] with isoniazid. Therefore, the BSA-Au NC-modified paper sensor can be used to detect the residual rifampicin in urine. Figure 1 presents a schematic of the preparation and detection strategy of the paper sensor for residual rifampicin in urine. The BSA-Au NC microplate paper sensor can be fabricated in three steps: printing patterns on the paper, melting wax to form hydrophobic barriers, and immobilizing the BSA-Au NCs in the reaction zones. After dropping the samples onto the paper sensor, the fluorescence images are captured by a digital camera and analyzed [38–40].

## Results and discussion

### Characterization of BSA-Au NCs

The BSA-Au NCs were developed using alkaline co-precipitation of gold salt with protein BSA [41], and they exhibited a sharp and intense emission peak centered at approximately 640 nm when excited at 480 nm (Additional file 1: Figure S1). The emission band centered at 640 nm began to appear after heating the reactants for approximately 10 min, and the emission intensity increased proportionally with 30 min of heating, although upon further heating, the intensity gradually decreased. Therefore, the optimal reaction time was 30 min for the synthesis of fluorescent BSA-Au NCs at 70°C at a pH of 11.5 (Additional file 1: Figure S1). The maximum fluorescence intensity of the emission peak of the BSA-Au NCs was obtained at an excitation wavelength of 480 nm (Additional file 1: Figure S2). Thus, the selection of 480 nm as the excitation wavelength with an emission peak centered at 640 nm and large Stokes shift (greater than 150 nm) eliminated the possibility of fluorescence self-quenching and measurement errors that might be caused by excitation light and scattering. The quantum yield of the prepared BSA-Au NCs was approximately 2% compared with that of Rhodamine 6G. In addition, the prepared BSA-Au NCs showed excellent stability over a wide range of pH values (2.0–10.0) (Additional file 1: Figure S3).

Solid-state Fourier transform infrared spectroscopy (FTIR) spectra were analyzed to investigate the surface structural changes after conjugating Au with BSA. The FTIR spectrum (Additional file 1: Figure S4) confirmed the presence of amide A, amide I, and amide II bands at 3,302.09 cm^−1^, 1,661.14 cm^−1^ and 1,530.14 cm^−1^, respectively, in pure BSA. The spectra of the BSA-Au NCs also showed three prominent bands corresponding to amide A, amide I, and amide II of the dipeptide at 3,297.41 cm^−1^, 1,658.18 cm^−1^ and 1,531.34 cm^−1^, respectively, which clearly demonstrated the dipeptide presence on the Au cluster surfaces. Additionally, a band at 2,961.20 cm^−1^ corresponding to C–H vibrations modes observed in pure BSA can also be observed in BSA-Au NCs. After adding rifampicin to the NCs, the FTIR spectra showed a considerable decrease in the broad band centered around 3,293.01. Although the characteristic peak of BSA at 3,293.01, 1,645.43 and 1,535.64 cm^−1^ were quite evident in the spectra (BSA-Au+R); peak broadening after the addition of rifampicin to the BSA-Au NCs could point towards the formation of hydrogen bonding between rifampicin and the BSA-Au NCs. The FTIR analysis illustrated that the surface structure of BSA remained intact even after the formation of BSA-Au NCs, implying that the BSA retained its reactive sites on the surface and could be further modified with new functional groups.

The size and shape of the BSA-Au NCs were investigated via electron microscopy. Additional file 1: Figure S5 presents the scanning transmission electron microscopy (STEM) (a) and high-resolution transmission electron microscopy (HRTEM) (b) images of the prepared BSA-Au NCs. The STEM and HRTEM images indicate that the NCs are well dispersed and the average diameter of the BSA-Au NCs is approximately 1.1 ± 0.1 nm as calculated from the images via ImageJ (Additional file 1: Figure S6).

Matrix-assisted lasers desorption/ionization mass spectroscopy (MALDI-MS) analysis was performed to reveal the number of Au atoms in the BSA-Au NCs. The samples were dialyzed against water using a 10-kDa-cutoff dialysis bag prior to MALDI-MS analysis. A broad peak for pure BSA was observed at *m/z* 65.4 kDa (Additional file 1: Figure S7a). After the reaction time was increased, the peaks for BSA-Au NCs became broader, and at a 30-min reaction time, we estimated that 18-atom Au NCs were formed (*m/z* 68.9) (Additional file 1: Figure S7b). The BSA undergoes partial denaturation upon heating at 70°C to expose amino residues, including histidine, cysteine, and tyrosine, which interact more strongly with Au^+^ (Au^3+^) to form NCs. Thus, one BSA conjugates with an 18-atom Au in the core to form BSA-Au NCs. The size of the formed Au cores is much smaller than that of BSA-Au_25_NCs prepared at room temperature for 12 h [20] because of the higher energy provided for the formation of BSA-Au NCs. On addition of 10 mM rifampicin to the BSA-stabilized Au NC, the MS spectra indicate that the characteristic peak of the BSA-Au NCs remained intact, thus indicating that leaching of Au had not occurred from the core of the BSA-Au NCs (Additional file 1: Figure S7c).

### Quenching mechanism of BSA-Au NCs by rifampicin

After adding rifampicin, the emission intensity of the NCs was greatly reduced. The MALDI-MS spectra (Additional file 1: Figure S7) of BSA-Au NCs both before and after adding rifampicin were quite similar, thus indicating the absence of any leaching of Au from the core of the BSA-Au NCs. To further prove that Au is not leached from the core of the BSA-Au NCs, an inductively coupled plasma mass spectrometry (ICP-MS) (data not shown) analysis was performed to determine the Au content of the purified BSA-Au NCs both in the presence and absence of rifampicin. The prepared aqueous solution of BSA-Au NCs had an Au content of 404.4 µg/mL, whereas upon interaction with rifampicin, 392.4 µg/mL of Au was detected in the aqueous solution of BSA-Au, revealing that the Au atoms in the NCs remained intact and were not filtered during dialysis.

**Figure 2:**
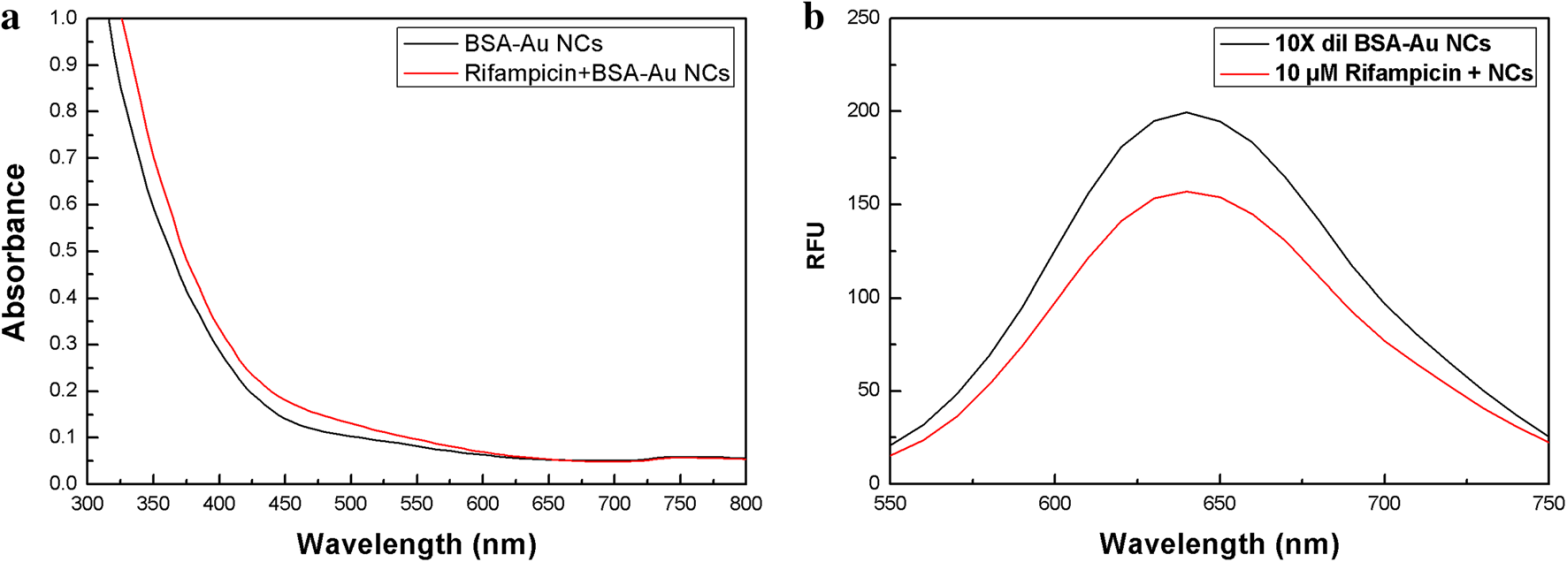
**a** Absorption spectra of BSA-Au NCs (0.1× dilution) in the absence and presence of 10 µM rifampicin. **b** Fluorescence emission spectra (excitation wavelength at 480 nm) of BSA-Au NCs (0.1× dilution) in the absence and presence of 10 µM rifampicin.

Au is conjugated with BSA through covalent bonding between the Au and thiol groups of the cysteine in BSA [41]. Rifampicin does not substitute the BSA and drive Au NC aggregation, which is evident from the MALDI-MS and ICP-MS data. Moreover, the absorption spectra indicate that aggregation did not occur, even after a long period of time following the addition of rifampicin to BSA-Au NCs (Figure 2a). However, the interaction of BSA and rifampicin has been previously reported [36]. Therefore, BSA-Au NC quenching by rifampicin (Figure 2b) may have been caused by the interaction of BSA and rifampicin, which changes the environment of the Au NCs. Changes in the absorption spectra of BSA-Au NCs in the presence of rifampicin (shown in Figure 2a) are similar to those reported for the interaction of BSA and rifampicin alone [36]. The absence of aggregated Au NCs without BSA protection corroborates the formation of structures similar to the rifampicin-RS-Au bonds.

Particle size measurements might offer a more sensitive tool for studying nanoparticle aggregation and hydrodynamic size distributions. BSA is a 66 kDa protein with a diameter of approximately 7–8 nm (data from Protein Data Bank). The dynamic light scattering (DLS) analysis could detect and measure the protein size directly (Additional file 1: Figure S8). The hydrodynamic size of BSA increases upon NC formation which can be conveniently observed via DLS. The diameter of the BSA-Au NCs increases to at least two times the diameter of the protein (BSA) molecule. After adding rifampicin to the NC, the hydrodynamic size of the BSA-Au NCs further increased significantly (Additional file 1: Figure S8), indicating the bonding of rifampicin to the surface of the NC.

### Fluorescence detection of rifampicin

**Figure 3:**
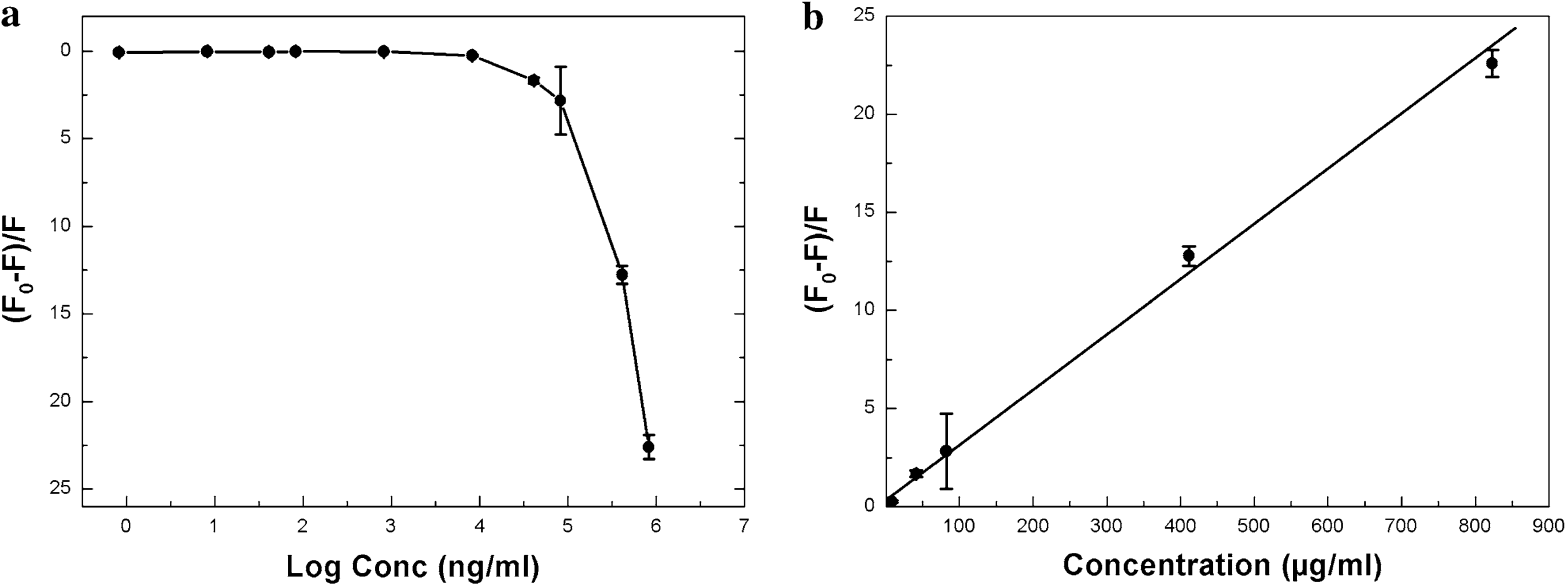
**a** Concentration-dependent quenching of BSA-Au NCs (0.1× dilution) by rifampicin. From higher to lower concentrations: normalized quenching at 823 µg/mL [(F_0_ − F)/F ~ 22.6 ± 0.68], 411 µg/mL [12.78 ± 0.5], 82 µg/mL [2.8 ± 1.93], 41 µg/mL [1.68 ± 0.16], 8 µg/mL [0.25 ± 0.6], 4 µg/mL [0.23 ± 0.008], 0.8 µg/mL [0.016 ± 0.009], 0.4 µg/mL [0.046 ± 0.031], 0.08 µg/mL [0.017 ± 0.016], and 0.004 µg/mL [0.06 ± 0.04] (each data point represents the average of three separate studies (n = 3), and the error bars denote the standard error of measurements within each experiment). **b** Plot of the linear region of the normalized decrease in fluorescence intensity of BSA-Au NCs (0.1× dilution) versus rifampicin concentration (each data point represents an average of three separate studies (n = 3); the error bars denote the standard error of measurements within each experiment). The excitation wavelength was set at 480 nm, and the emission wavelength was 640 nm.

With the inherent fluorescence of BSA-Au NCs and its high sensitivity, the synthesized NCs could be used to detect rifampicin via rapid fluorescence quenching. The fluorescence intensity of the BSA-Au NCs decreased proportionally with increases in rifampicin concentration (Additional file 1: Figure S9). As shown in Figure 3a, a nearly linear relationship was observed between the normalized decrease in fluorescence intensity (F_0_ − F)/F and concentration of rifampicin over a range from 0.5 to 823 µg/mL. However, the normalized decrease in fluorescence intensity reached a plateau when the concentration of rifampicin fell below 0.5 µg/mL.

Plotting of the normalized decrease in fluorescence intensity (F_0_ − F)/F versus the log of the rifampicin concentration resulted in a linear calibration graph over the concentration range from 0.5 to 823 µg/mL (correlation coefficient: R^2^ = 0.9945) with a limit of detection (LOD) of 0.07 µg/mL (Figure 3b). The limit of quantification (LOQ) was 0.2 µg/mL. The reproducibility of the sensing system was established by performing three independent measurements using 20.6 µg/mL rifampicin with a relative standard deviation of 4.22% (R^2^ = 0.9948). This measurement was conducted to verify the reliability of the proposed method, which presents high sensitivity and notably low relative standard deviations.

### Selectivity of BSA-Au NCs toward detecting rifampicin

**Figure 4:**
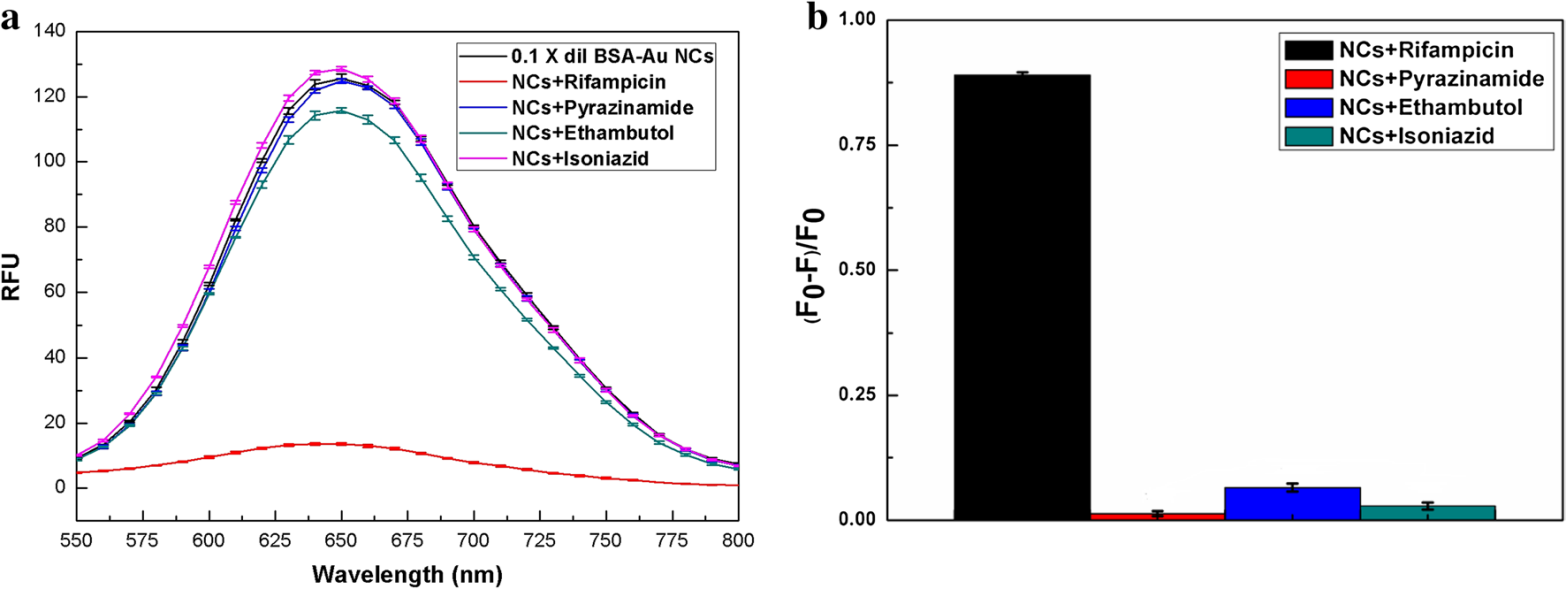
**a** Fluorescence emission spectra of BSA-Au NCs (0.1× dilution) in the presence of primary TB drugs (100 µM each in the final concentration). At 640 nm (emission wavelength): BSA-Au NCs (fluorescence intensity = 123.74 ± 1.56), NCs + rifampicin (83 µg/mL) (13.67 ± 0.33), NCs + pyrazinamide (12.3 µg/mL) (121.95 ± 0.69), NCs + ethambutol (27.7 µg/mL) (114.27 ± 1.28) and NCs + izoniazid (13.7 µg/mL) (127 ± 0.69) (each data point represents the average of three separate studies (n = 3), and the *error bars* denote the standard error of measurements within each experiment). **b** Comparison of the normalized decrease in fluorescence intensity of BSA-Au NCs (0.1× dilution) in the presence of primary TB drugs (100 µM each in final concentration). At 640 nm (emission wavelength): normalized quenching of NCs + rifampicin [(F_0_ − F)/F_0_ ~ 0.89 ± 0.006], NCs + pyrazinamide [0.013 ± 0.005], NCs + ethambutol [0.065 ± 0.008] and NCs + izoniazid [0.028 ± 0.007] (each data point represents the average of three separate studies (n = 3), and the error bars denote the standard error of measurements within each experiment). [In this work, (F_0_ − F)/F_0_ = 1 indicates complete quenching and (F_0_ − F)/F_0_ = 0 indicates no quenching]. The excitation wavelength was set at 480 nm, and the emission wavelength was 640 nm.

We tested the selectivity of BSA-Au NCs for sensing rifampicin in the presence of other antibiotics (INZ, E and P) used collaterally with rifampicin for the treatment of TB. The fluorescent responses of BSA-Au NCs with all four antibiotics were monitored and compared with that of rifampicin. Figure 4a shows that the addition of rifampicin at a final concentration of 100 µM (83 µg/mL) resulted in a nearly 90% decrease in the fluorescence intensity of the BSA-Au NCs, whereas a limited decrease in fluorescence intensity was observed for P (1.3%), INZ (2.8%) and E (6.5%) at the same molar concentration (Figure 4b). The clinical effectiveness of rifampicin as a primary drug for TB can mainly be attributed to 4-methyl-1-piperazinaminyl substitution on rifampicin. Generally, antibiotics with piperazine moiety exhibit good efficacy as therapeutic agents [42–44]. Therefore, we tested the specificity of BSA-Au NCs toward detecting rifampicin using antibiotics with piperazine functional group such as ciprofloxacin [42], buspirone [43], ipsapirone [44] (Additional file 1: Figure S10a). In addition, we also measured the fluorescence responses of some commonly used antibiotics and drugs (Additional file 1: Figure S10b). The average amount of residual rifampicin present in the patients’ urine is in the range of 45–55 µg/mL [45]. Our results showed that at a final concentration of 50 µg/mL of rifampicin a nearly 60% decrease in the fluorescence intensity of the BSA-Au NCs was measured, whereas only a limited decrease in fluorescence intensity (less than 10%, p < 0.001) was observed for all the other antibiotics and drugs at the same concentration. Therefore, BSA-Au NCs can be used for preferential sensing of rifampicin among other antibiotics administered at the same time. Additionally, the prepared BSA-Au NCs were freeze-dried and stored for 3 months before dispersion for the same experiment (Additional file 1: Figure S11). No drastic changes were observed, even after storage, indicating the excellent stability of the BSA-Au NC.

### Tolerance to potentially interfering ions

In a real urine sample, the presence of ions might interfere with rifampicin sensing. To evaluate the tolerance level to these ions, solutions containing specific ions were tested until the interfering substances had a minimal effect on fluorescence quenching of the BSA-Au NCs (less than 5% change). The results from the potentially interfering substances are presented in the Additional file 1: Table S1 together with the typical ion concentrations found in the real urine sample. The selective interaction of BSA-rifampicin was stronger than other ionic interferences. At a tenfold dilution, the interference from ions in urine is negligible.

### Detection of rifampicin in spiked urine

After determining that the BSA-Au NCs have excellent sensitivity and specificity in sensing rifampicin, the next step was to determine the concentration of rifampicin in urine using BSA-Au NCs. Generally, evaluating the intake dosage of rifampicin for a patient requires urine collection over a long period of time (8–24 h) after ingestion of the drug followed by analysis in a laboratory equipped with sophisticated instrumentation. In the urine sample, the average indicative levels of rifampicin intake ranged from 45 to 55 µg/mL [45]. In our experiment, various concentrations of rifampicin were spiked into 10 mL of fresh urine collected from a healthy individual. A tenfold dilution of the urine sample containing rifampicin was used for the quantitative analysis via fluorescence quenching (turn-off sensing mechanism). The recovery of rifampicin was estimated at greater than 85% in the concentration range normally present in actual urine samples (i.e., 5–60 µg/mL). Additional file 1: Table S2 presents the comparative results for determining rifampicin levels using other assays.

### BSA-Au NCs immobilized on a paper platform for detection of rifampicin in spiked urine

**Figure 5:**
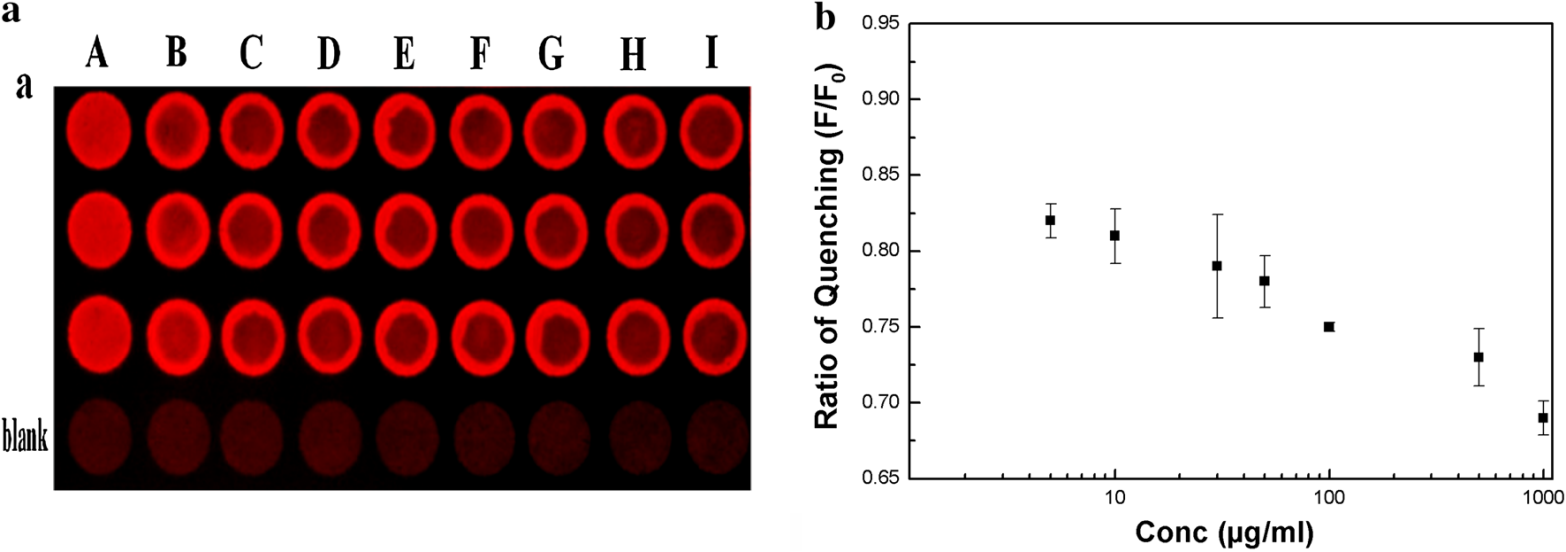
**a** Test paper for the detection of rifampicin after modification (*A*) with BSA-Au NCs under UV light. The tenfold diluted urine samples with original rifampicin concentrations are as follows: (*B*) 0.5 µg/mL (fluorescence quenching ratio = 91% ± 1); (*C*) 5 µg/mL (82% ± 1.1); (*D*) 10 µg/mL (81% ± 1.8); (*E*) 30 µg/mL (79% ± 3.4); (*F*) 50 µg/mL (78% ± 1.7); (*G*) 100 µg/mL (75% ± 0.3); (*H*) 500 µg/mL(73% ± 1.9); and (*I*) 1000 µg/mL(69% ± 1.1). **b** Change in the fluorescence quenching ratio of the embedded BSA-Au NCs versus rifampicin concentration on the paper sensor (each data point represents an average of three separate studies (n = 3) in three different wax-printed 96-microplate paper platforms; three measurements were taken in each micro-well, and the error bars denote the standard deviation of the reading).

Disposable sensors are desirable in point-of-care applications. Therefore, we immobilized BSA-Au NCs on a wax-printed paper-based platform to conduct real-time monitoring of rifampicin in urine. The urine samples were collected from patients at the Tri-Service General Hospital in Taipei, Taiwan. The patients who contributed urine samples signed informed consent forms as required by the regulations of the Institutional Review Board of the Tri-Service General Hospital of Taipei, Taiwan. Initially, 30 µL of the prepared BSA-Au NCs were dropped in each well of the 96-well wax-printed paper platform and dried. The changes observed after the BSA-Au NCs were immobilized on the paper under ultraviolet (UV) light are shown in Figure 5a(A). The images were captured using a digital camera (Olympus, E-330), and the images were processed by MetaMorph software using only the ‘red’ color and center portion of each well to calculate the effective fluorescence intensity quenching ratios. The area without BSA-Au NCs (blank) was used as the background. Pale red fluorescence was clearly observed after immobilization of the BSA-Au NCs on the paper platform. Different amounts of rifampicin were spiked in the collected urine samples, and tenfold diluted urine samples were used for the quantitative analysis.

Increases in rifampicin concentration increased the fluorescence quenching of the imbedded BSA-Au NCs in the wax-printed paper sensor. Each colorimetric assay was performed using a total volume of 30 µL prepared BSA-Au NCs, and the evaluation was performed with a total of 30 µL spiked urine containing different concentrations of rifampicin. The amount of fluorescence quenching was lowest with spiked urine containing 0.5 µg/mL rifampicin (Figure 5a(B)), followed by that of 5 µg/mL (Figure 5a(C)), 10 µg/mL (Figure 5a(D)), 30 µg/mL (Figure 5a(E)), 50 µg/mL (Figure 5a(F)), 100 µg/mL (Figure 5a(G)), 500 µg/mL (Figure 5a(H)) and 1000 µg/mL (Figure 5a(I)). Our detection scheme can be further simplified via the use of a mobile imaging device that can determine the ratio of BSA-Au NCs quenching by capturing the image intensity and perform analyses through an installed application [38–40]. This process eliminates the need to install an image analyzer at the site of rifampicin detection. Figure 5b plots the data obtained from three different paper sensors with three independent data points for each concentration of rifampicin. Our results show that even the minimum therapeutic dosage [45] (5 µg/mL) of rifampicin in the urine can be observed with the paper sensor platform. Therefore, the actual amount [45] (45–55 µg/mL) of rifampicin present in the urine sample can be monitored easily using this methodology. The results obtained for all data points were similar when the tests were performed with different paper platforms (Additional file 1: Table S3). This paper-based assay offers numerous advantages over solution-phase fluorescent probes, including its low cost, rapidity, simplicity, robustness and ability to perform real-time monitoring of samples.

## Conclusion

We have developed a robust, cost-effective and portable point-of-care medical diagnostic platform for the detection of rifampicin in urine based on the ability of rifampicin to quench the fluorescence of immobilized BSA-Au NCs on wax-printed paper. Water-soluble Au NCs were synthesized within 30 min at 70°C using BSA as a template. The prepared BSA-Au NCs possessed high fluorescence emission intensity and stability. The BSA-Au NCs showed remarkable selectivity towards rifampicin over other TB drugs. The fluorescent property of the BSA-Au NCs was retained after immobilization on paper, and the quenching of fluorescence by rifampicin in urine can be observed by the naked eye under UV irradiation. We demonstrated that a mobile imaging device can be used in data acquisition to capture the image intensity as well as data analysis to quantify the rifampicin. Therefore, the semi-quantitative assay presented here could be useful for monitoring drug intake in TB patients on a regular basis and can act as a first line of detection to complement current techniques used to detect rifampicin in urine. The paper-based assay can also be used to detect other specific analytes via surface modification of the BSA in the BSA-Au NCs and has the potential for use in the monitoring of other diseases.

## Methods

### Materials

BSA was purchased from Sigma-Aldrich (A7906-10g). Hydrogen tetrachloroaurate(III) trihydrate (HAuCl_4_) and isonicotinic acid hydrazide were obtained from Alfa Aesar. Rifampicin was purchased from Sigma Aldrich (R3501-1g), pyrazinamide was obtained from Acros Organics, and ethambutol dihydrochloride was obtained from LKT Laboratories, Inc. Sodium hydroxide was obtained from Riedel–de Haen. All of the solvents and chemicals were used without further purification. Aqueous solutions were prepared using deionized water (DI) with a resistivity of 18.20 MΩ cm.

### Instrumentation

A SpectraMax M2 (Molecular Devices) spectrophotometer was used to measure the visible ultraviolet (UV–Vis) absorbance of the protein–Au NCs and their fluorescence intensities with an excitation wavelength of 480 nm and emission wavelength of 640 nm. The FTIR (PerkinElmer Spectrum 100 FT-IR Spectrometer) spectra were recorded to determine the structural changes before and after conjugation of Au with BSA. The STEM and TEM (JEOL JEM-2100 Field Emission Transmission Electron Microscope) images were used to determine the size distributions and measure the dispersibility of the prepared nanoparticles. The MALDI-MS (MALDI TOF–TOF, Bruker, UltraflXtreme) analysis was applied to reveal the number of Au atoms in the BSA-Au NCs. The ICP-MS (Thermo X-Series II) analysis was applied to determine the Au content of the purified BSA-Au NCs both in the presence and absence of rifampicin. A 90 Plus particle size analyzer (BIC) was used for the size distribution studies.

### Synthesis of BSA-stabilized gold nanoclusters

According to the reported protocol [41], 5 mL of an aqueous solution of HAuCl_4_ (10 mM) was added to 5 mL BSA (50 mg/mL in water) under vigorous stirring for 30 s. Next, the pH of the solution was adjusted to approximately 11.5 through the addition of NaOH (0.5 mL, 1 M). The reaction was conducted at 70°C and reached its maximum intensity after 30 min of reaction time with completion of the BSA-Au conjugation (Additional file 1: Figure S1), wherein the solution turned from pale yellow to dark red, thus indicating the formation of BSA-Au NCs. The BSA-Au NCs were freeze-dried and stored at 4°C and did not precipitate, even 3 months after re-dispersion in water. The use of the BSA-template-mediated green synthesis route for the production of fluorescent BSA-Au NCs through the proposed mechanism [41] considerably reduced the synthesis time and eliminated the need for further purification of toxic reagents.

### Rifampicin detection procedure

Stock solutions (10 mM) of isoniazid (INZ), rifampicin (R), ethambutol (E) and pyrazinamide (P) were prepared. Subsequently, 1 mL of the prepared BSA-Au NCs and 100 µL of the antibiotic solution were added to a bottle, and the volume was adjusted to 10 mL to a final antibiotic concentration of 100 µM. The fluorescence intensities were recorded immediately with an excitation wavelength of 480 nm and emission wavelength of 640 nm. The calibration curve for rifampicin was prepared according to the normalized decrease in fluorescence intensity, which is defined as (F_0_ − F)/F, where F_0_ and F are the maximum emission intensities of the BSA-Au NC system in the absence and presence of rifampicin, respectively.

### Characterization of BSA-Au NCs

Field emission transmission electron microscopy was used on the prepared BSA-Au NCs to calculate the size distribution at 200 kV. The samples were prepared by applying the diluted solution (20-fold dilution from the prepared solution) onto formvar stabilized with a carbon TEM grid (200 mesh size; TED PELLA) followed by drying at room temperature. The MALDI-MS analysis was used to characterize the BSA conjugation with Au atoms. The ICP-MS analysis was applied to determine the Au content of the BSA-Au NCs both in the presence and absence of rifampicin.

### BSA-Au NCs on paper platform synthesis

Advantec chromatography paper sheets (grade No.1, Toyo Roshi Kaisha Ltd) were cut into standard A4 sheets that directly fit into the feed tray of a commercial solid ink printer (ColorQube™8570, Xerox Corporation). The ink printers were capable of printing patterns that serve as hydrophobic barriers after proper heating. Paper mircroplates were designed with a standard 96-well format. All of the measurements followed the “Corning Microplate Selection guide” (Corning Inc., USA). The printed pattern of a 96-well plate was placed on a hot plate (Corning PC-420d, Corning Inc.) at 150°C for 2 min [12, 13], which melted the wax and created a hydrophobic barrier because of vertical percolation of the wax in the paper. Each well was imbued with 30 µL of the prepared BSA-Au NCs and allowed to dry at 40°C for 30 min. The microplates were stored in the dark at room temperature (25–35°C).

## Additional file

Additional file 1: Supplementary Data.

## Abbreviations

BSA: bovine serum albumin
TB: tuberculosis
R: rifampicin
INZ: isoniazid
E: ethambutol
P: pyrazinamide
BSA-Au NCs: bovine serum albumin-stabilized gold nanoclusters
NCs: nanoclusters
