# Supplementary material for: Detection of residual rifampicin in urine via fluorescence quenching of gold nanoclusters on paper
Source: J Nanobiotechnology. 2015 Jun 26;13:46. doi: 10.1186/s12951-015-0105-5 (PMC4482266; doi:10.1186/s12951-015-0105-5)
Supplement: Additional file 1: — Supplementary Data. [file 12951_2015_105_MOESM1_ESM.docx]

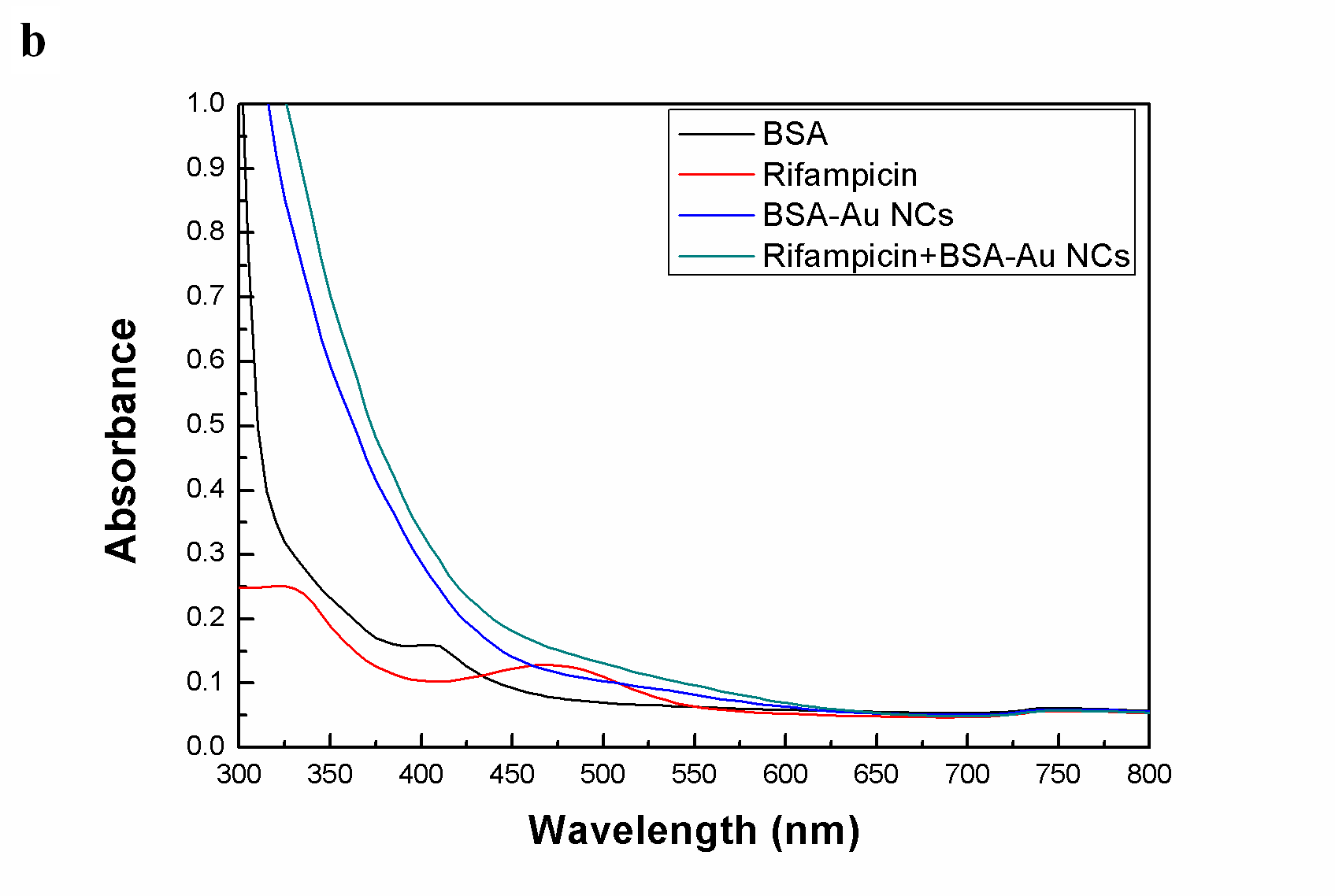


Supplementary 1: (a) Growth Spectra of BSA-Au at 70ºC. Inset- plot of time (Mins) vs integrated fluorescent intensity at different time intervals. (b) Absorption spectra of BSA (2.5 mg/mL), BSA-Au NCs (0.1X dilution), Rifampicin (10 µM) and Rifampicin (10 µM) + BSA-Au NCs (0.1X dilution).





Supplementary 2: Emission spectra of BSA–AuNCs at different excitation wavelength





Supplementary 3: pH dependence of BSA-Au NCs (0.1X dilution) in 5mM phosphate buffer. All other conditions same as synthesis in 30 min figure S1 (a).


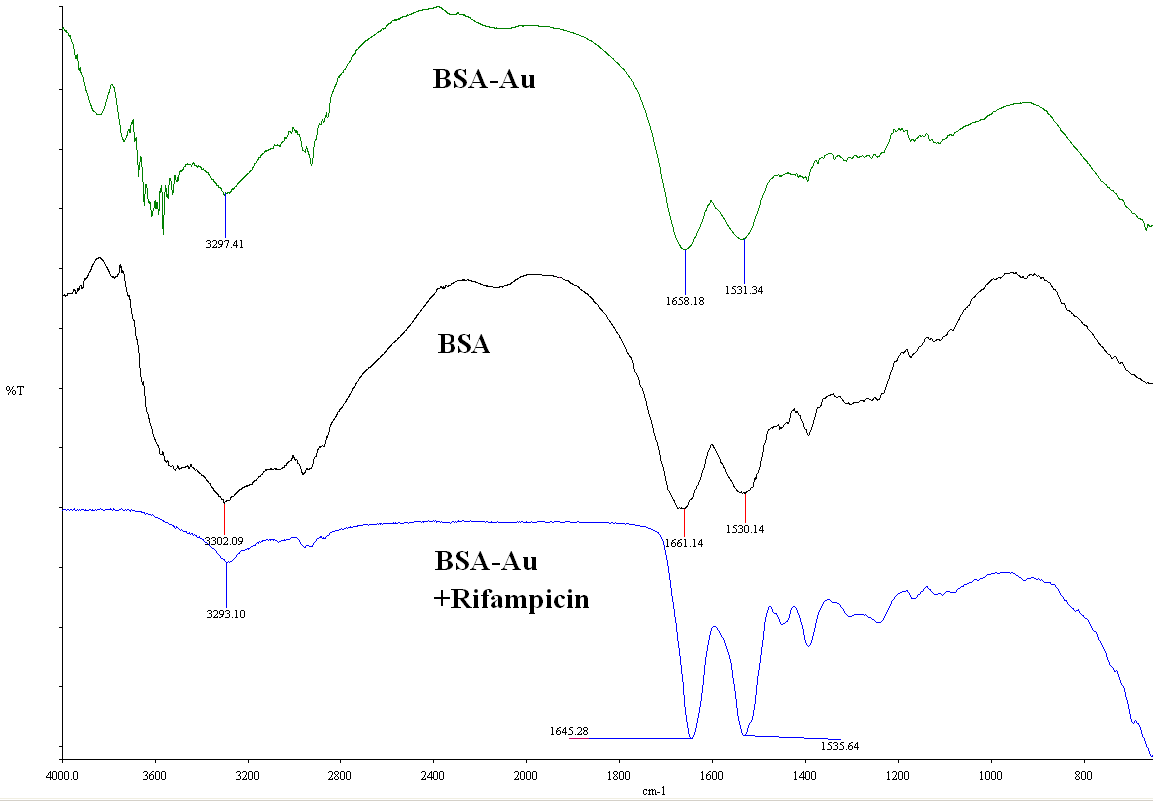


Supplementary 4: FTIR spectra of BSA-Au (as prepared), BSA and BSA-Au+Rifampicin (from bottom to top).


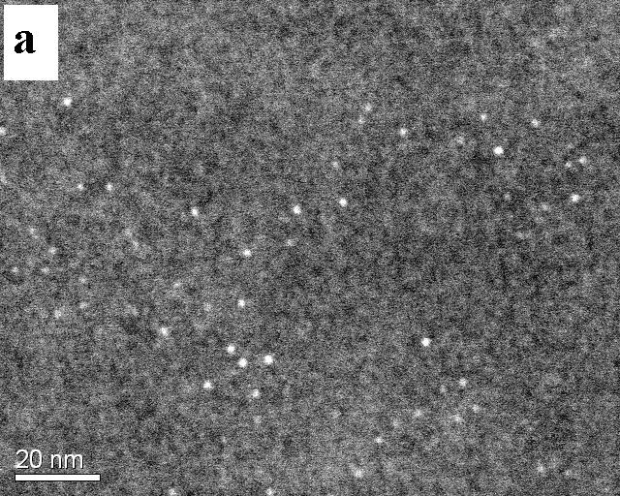

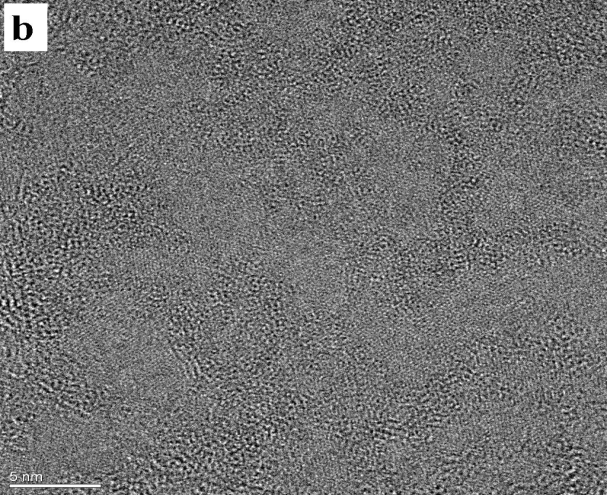


Supplementary 5: (a) STEM image of Supplementary 5: (b) HRTEM image of BSA-Au NC. BSA-Au NC





Supplementary 6: Size distribution of BSA AuNCs as calculated from images using ImageJ.


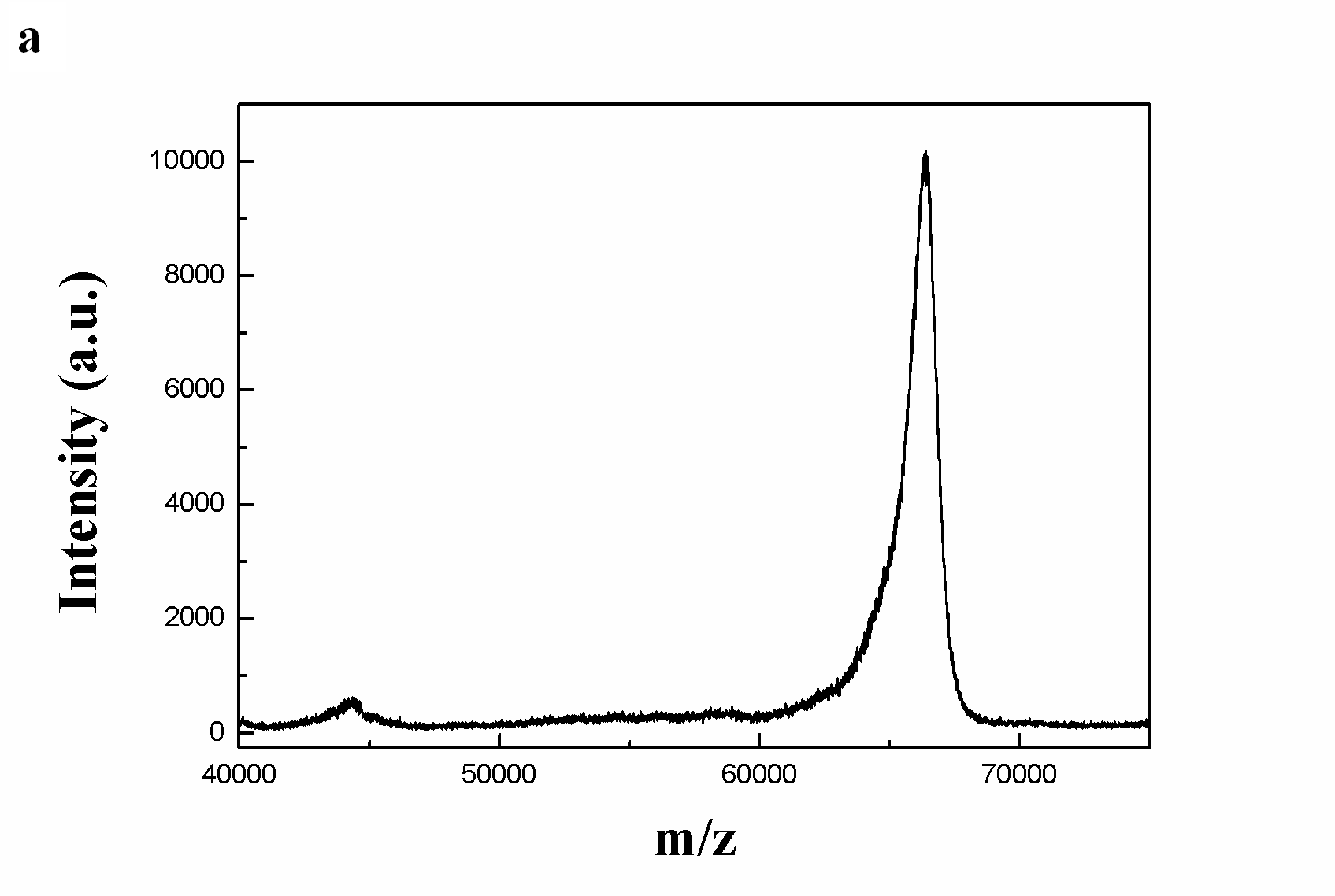


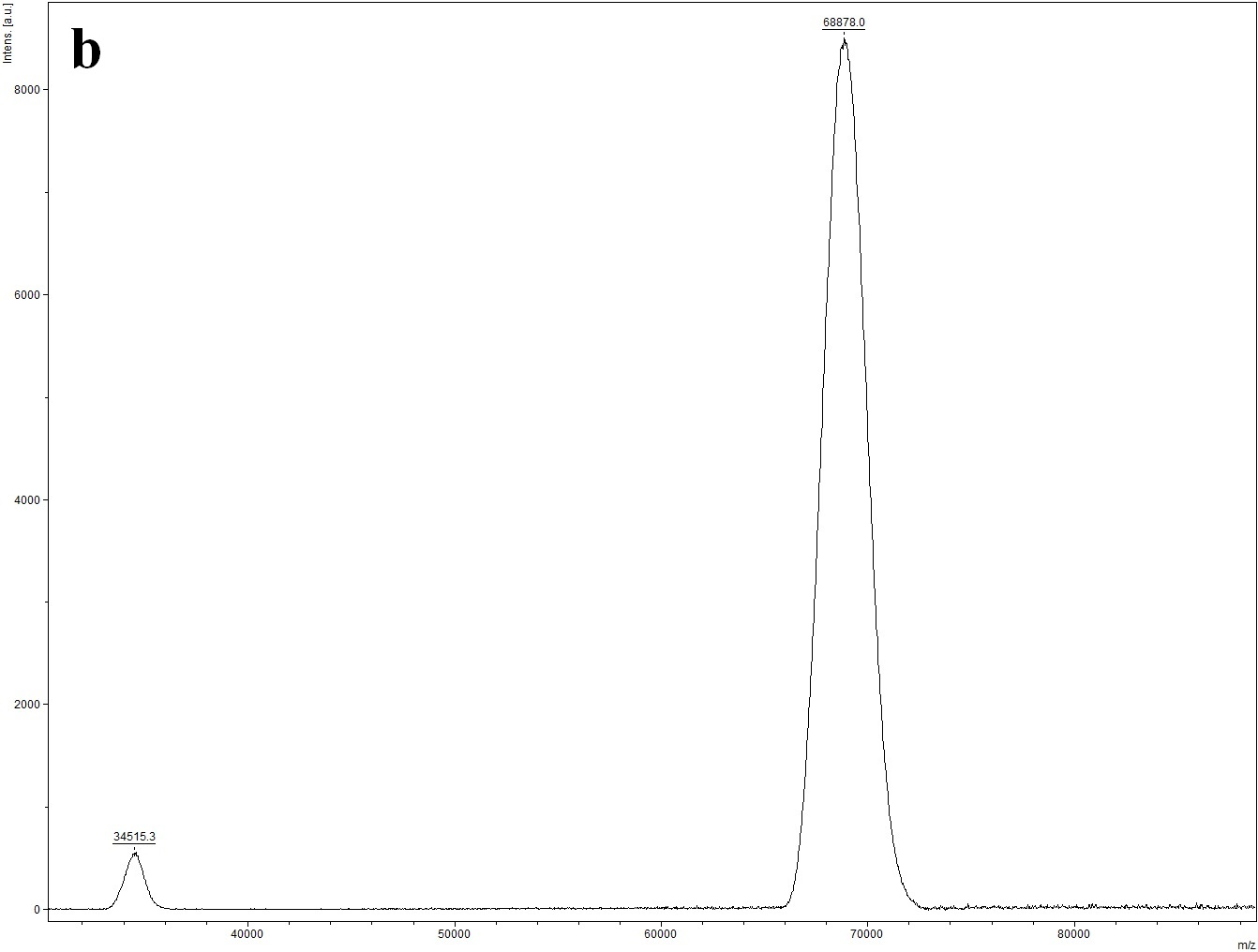


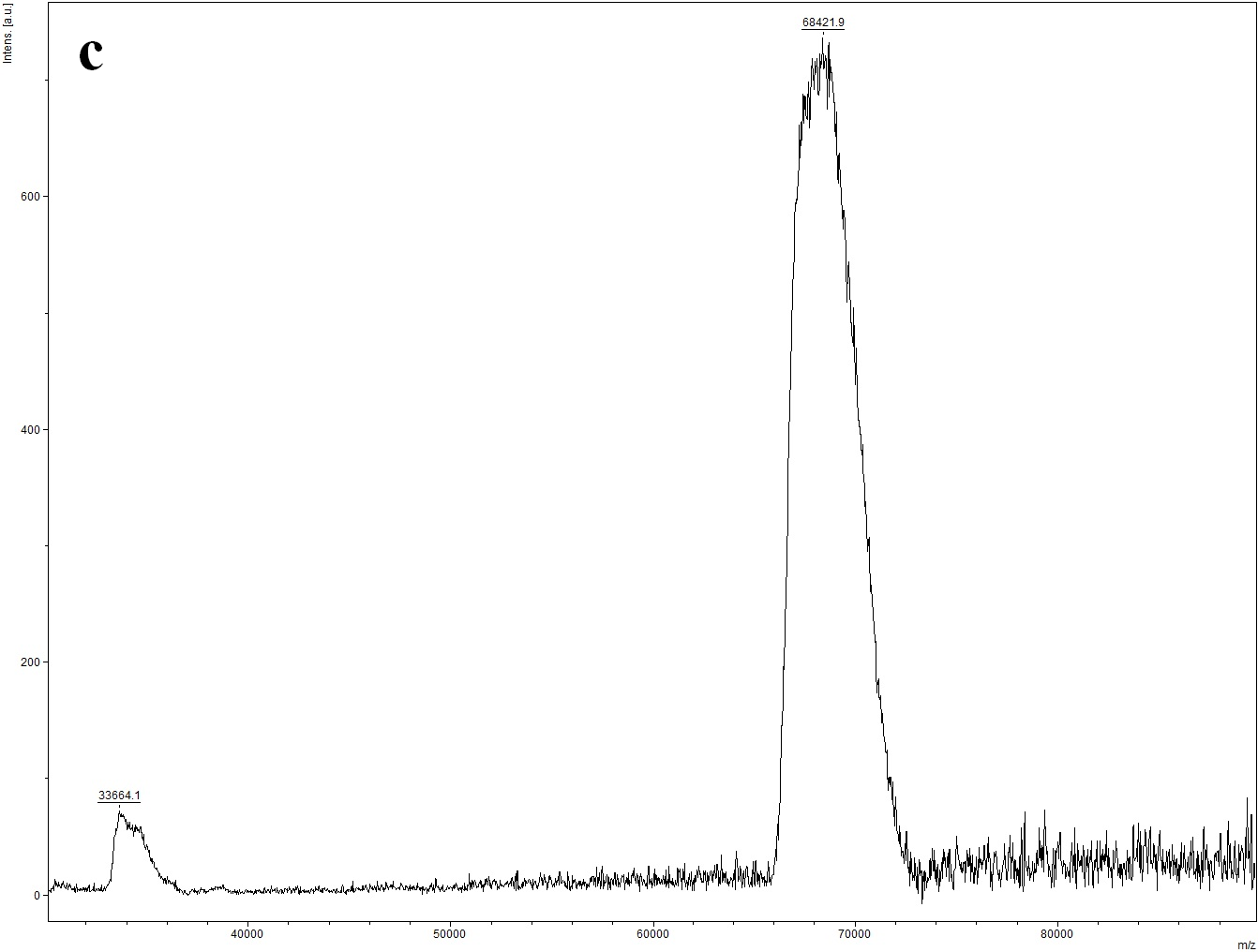


Supplementary 7: MALDI-MS spectra of solutions a. BSA (3.7 µM), b. BSA-Au NCs (0.1 X dilution) c. BSA-Au NCs and 10 µM rifampicin (0.01 X dilution).


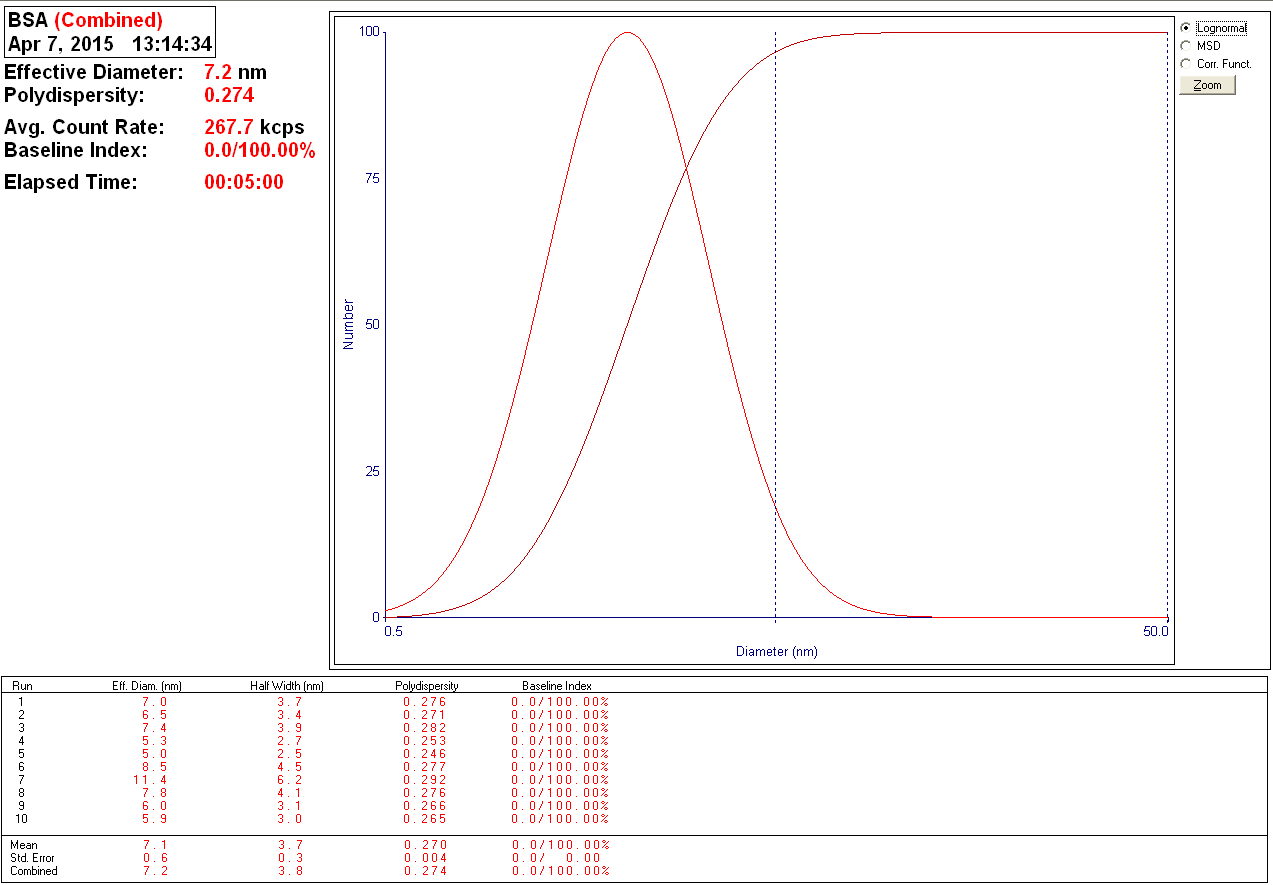

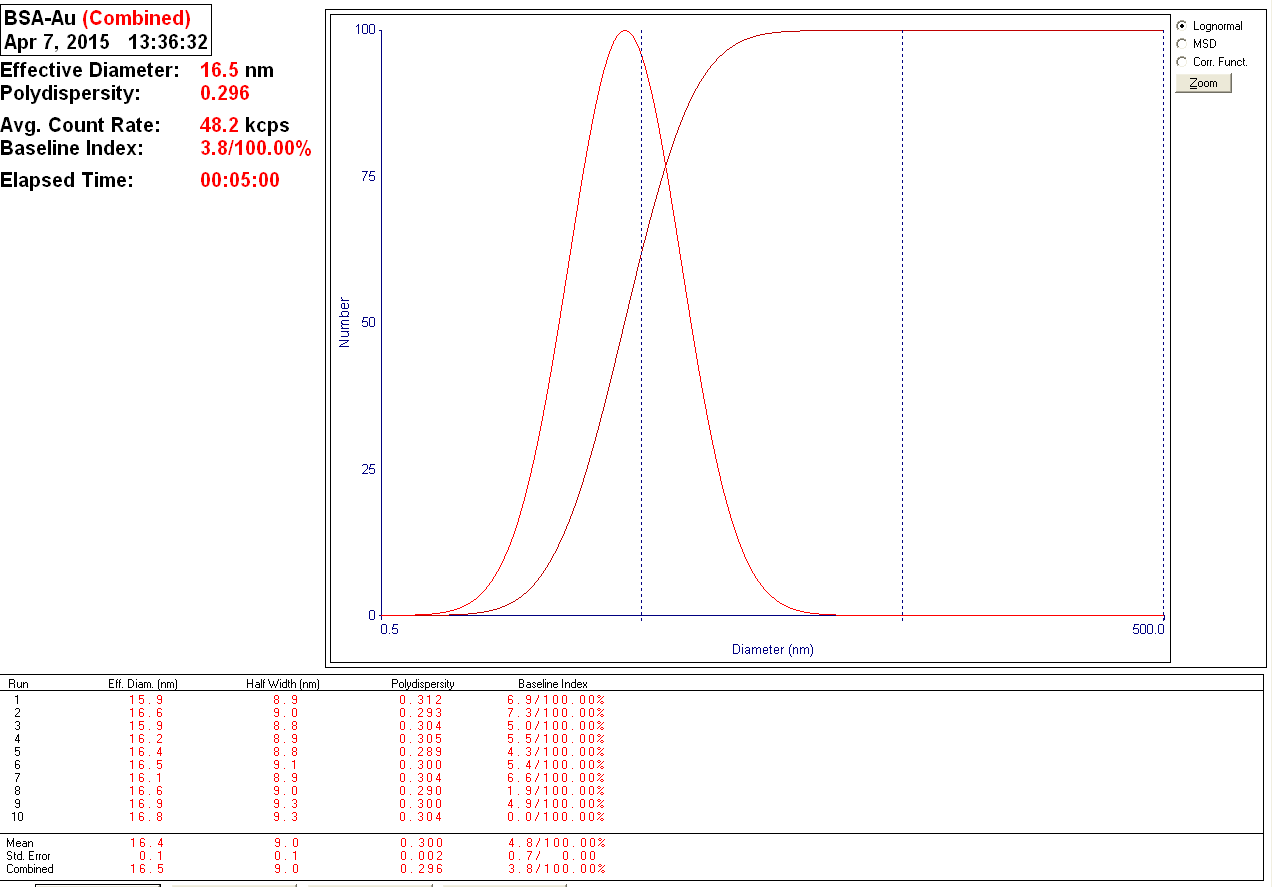

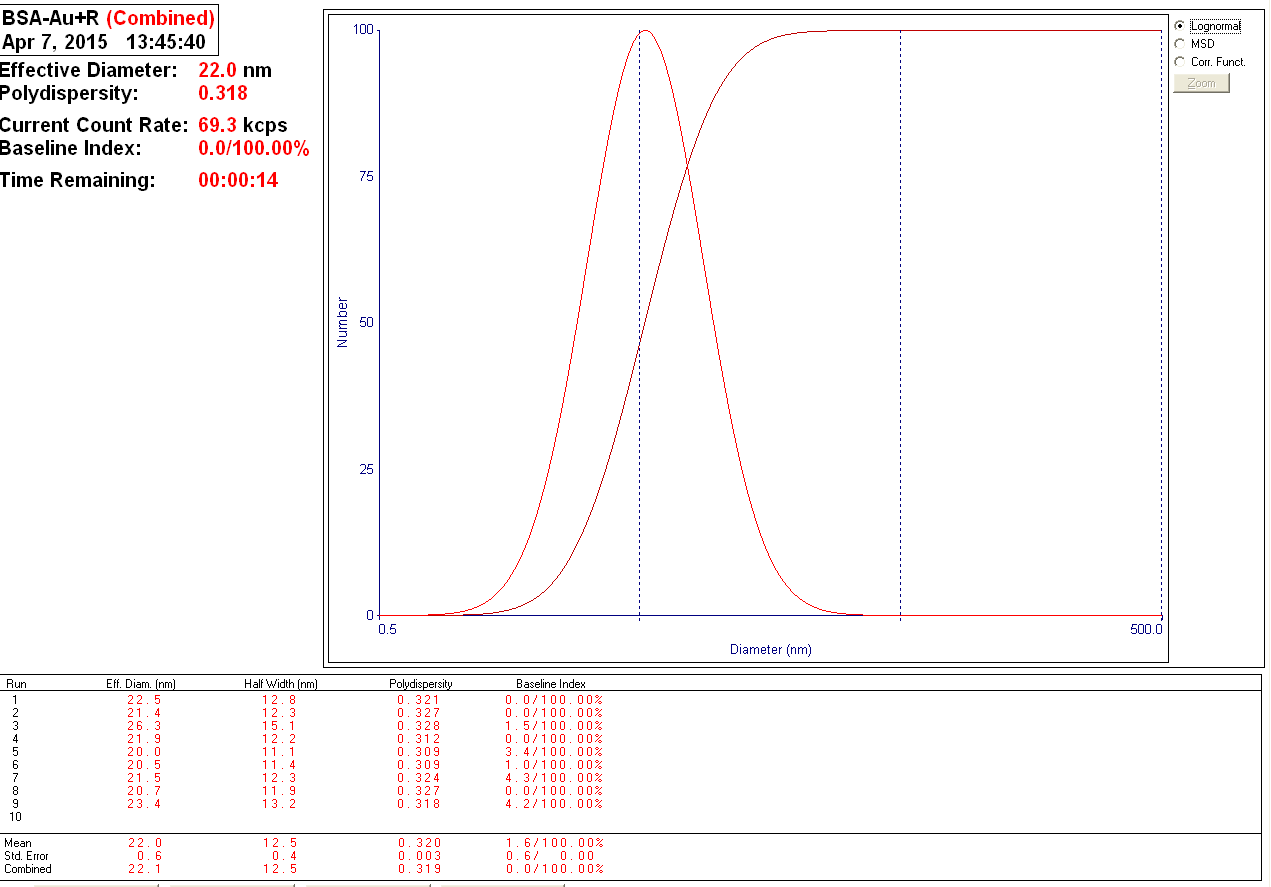


Supplementary 8: Dynamic Light Scattering (Zeta particle size analyzer) study of pure BSA, BSA-Au NCs and BSA-Au + Rifampicin.





Supplementary 9: Decrease in the fluorescence emission spectra of BSA-Au NCs in the presence of increasing concentrations of rifampicin. The excitation wavelength was set at 480 nm and emission was at 640 nm.


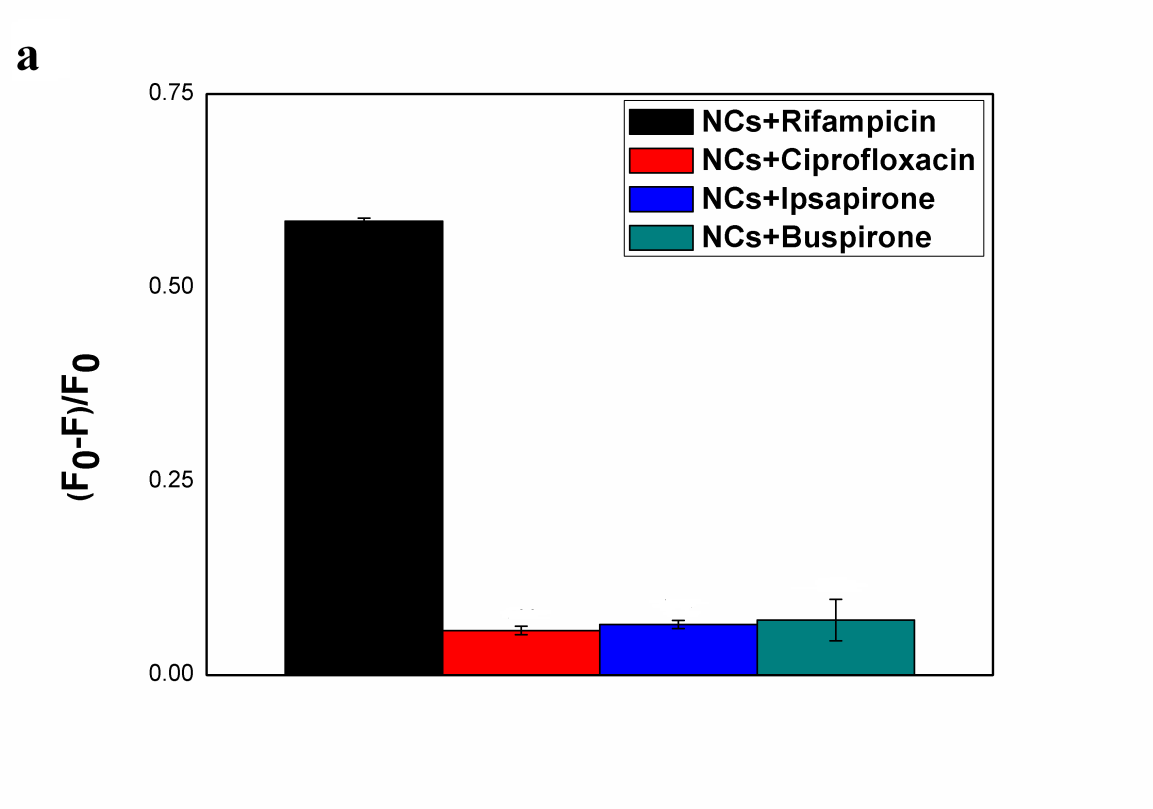


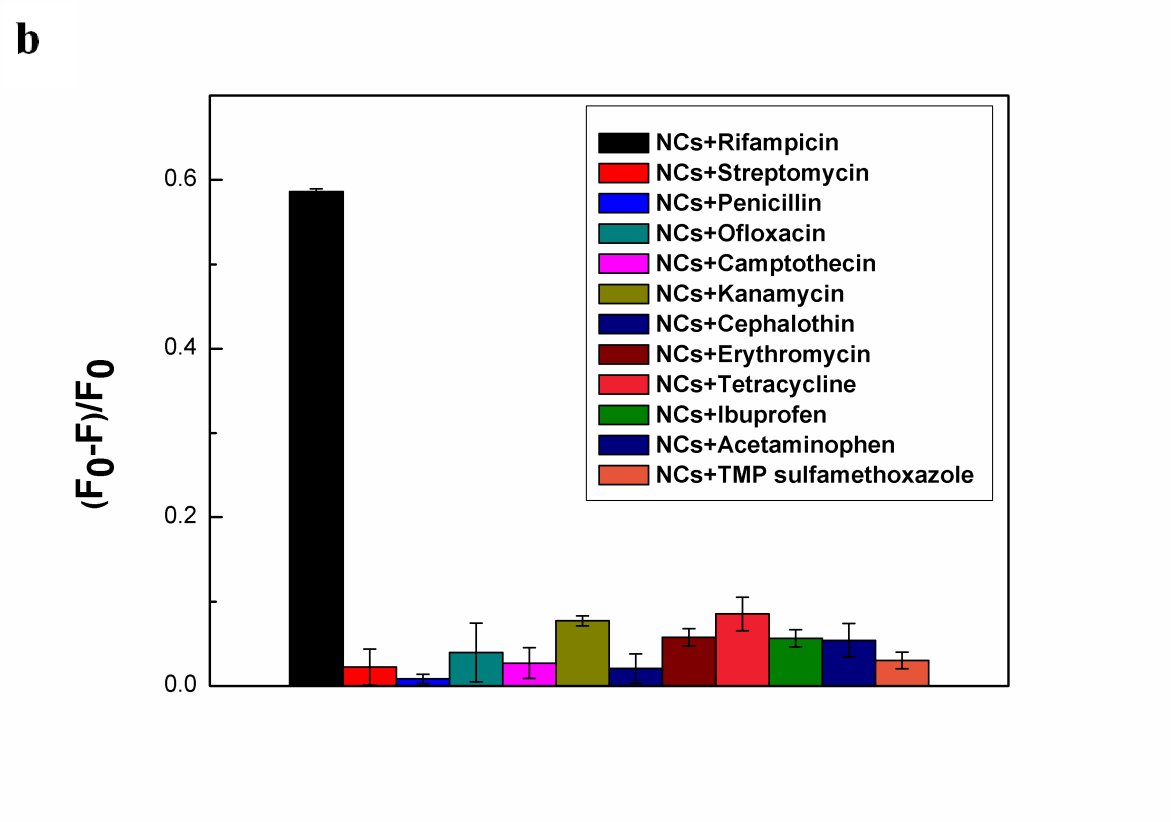


Supplementary 10: (a) Fluorescence emission spectra of BSA-Au NCs (0.1X dilution) in the presence of piperazine moiety containing drugs (50 µg/ml each in the final concentration). (b) Fluorescence emission spectra of BSA-Au NCs (0.1X dilution) in the presence of some commonly used antibiotics (p<0.001) (50 µg/ml each in the final concentration).





Supplementary 11: Fluorescence emission spectra of BSA-Au NCs (0.1X dilution) in the presence of primary TB drugs (100 µM each in final concentration) BSA-Au NCs was crystallized and re-dispersed in water after 3 months, before performing this experiment. The excitation wavelength was set at 480 nm and emission was at 640 nm.


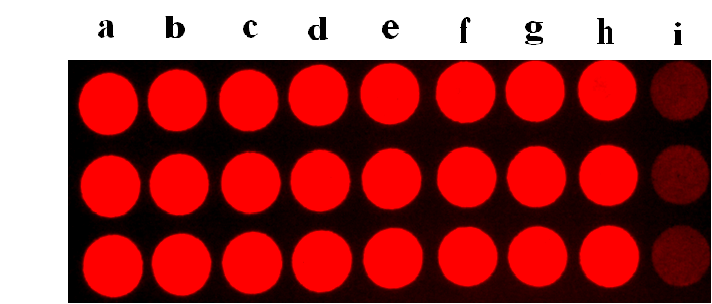


Supplementary 12: Test paper for the detection of rifampicin after modification (a) with 30 µl BSA-Au NCs under UV light. (b) 5 µl water (99.3%). Urine diluted 10-fold (c) 5 µl (97.8%), (d) 7.5 µl (97.6%), (e) 10 µl (97.6%), (f) 12.5 µl (97.4%), (g) 15 µl (97.4%), (h) 20 µl (94.5%) & (i) Blank.

`
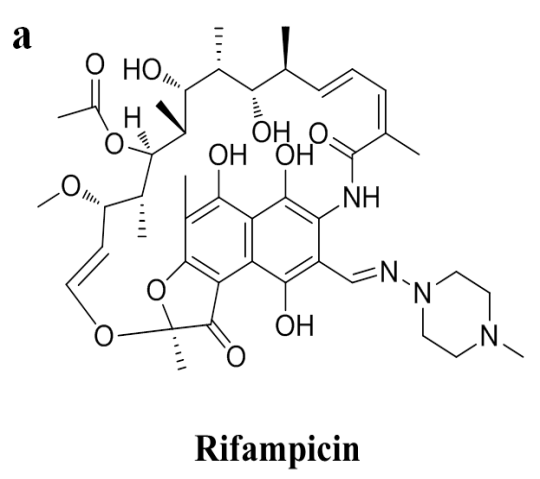

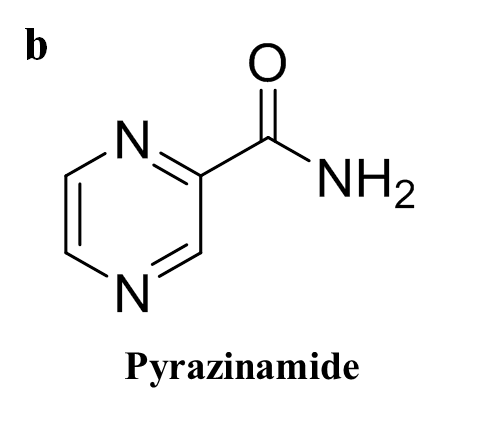


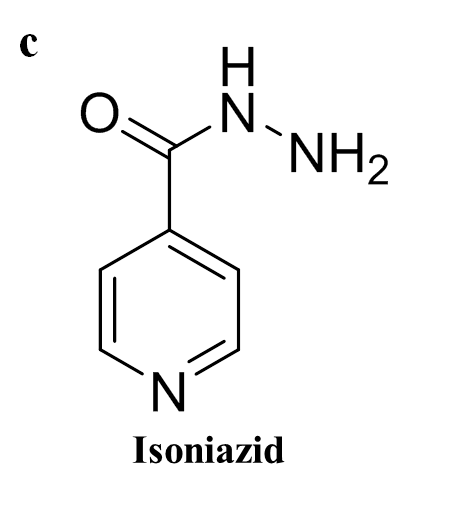

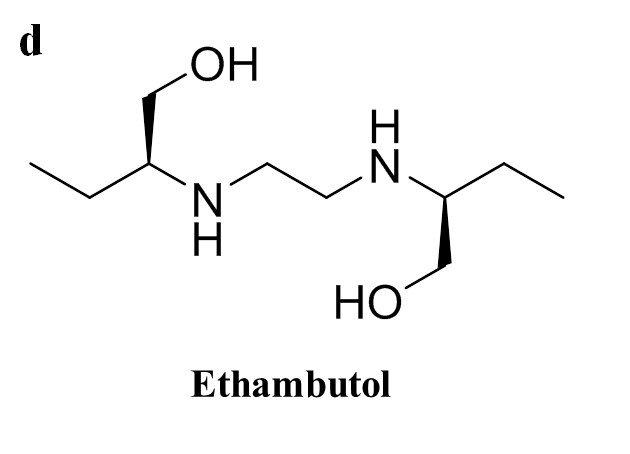


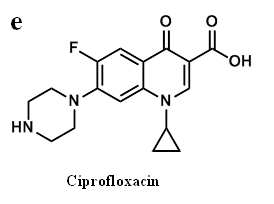

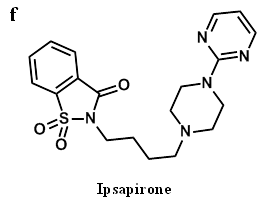


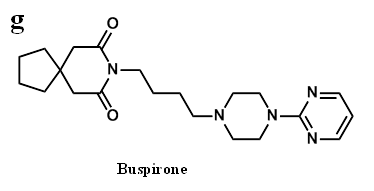


Supplementary 13: Sturctures of (a) Rifampicin (b) Pyrazinamide (c) Isoniazid (d) Ethambutol (e)Ciprofloxacin (f) Ipsapirone (g) Buspirone.

Table S1:

**Ions Concentration Concentration in Actual Concentration in**

**Tolerated by Au NCs Urine (approx) Study (10X dil Urine)**

Al^3+^ 134.9 µg/mL <10 ng/mL 1 ng/mL

K^+^ 391 µg/mL 2.6 mg/mL 261 µg/mL

Na^+^ 1.2 mg/mL 8.4 mg/mL 1 mg/mL

Mg^2+^ 121.5 µg/mL 0.1 mg/mL 10 µg/mL

Pb^2+^ 1 µg/mL 0.1 µg/mL 12 ng/mL

Cu^2+^ 0.3 µg/mL 0.02 µg/mL 2 ng/mL

Zn^2+^ 0.3 µg/mL 1.5 µg/mL 154 ng/mL

Fe^2+^ 279.2 µg/mL 0.2 µg/mL 21 ng/mL

Mn^2+^ 0.3 µg/mL 0.3 µg/mL 35 ng/mL

SO_4_^2-^ 480 µg/mL 0.8 mg/mL 77 µg/mL

Cl^-^ 1.8 mg/mL 23.3 mg/mL 2 mg/mL

Table S2:

**Sample Method Detection Limit Reference**

Rifampicin Spectrophotometry 0.4 µg/mL Mansilla A.E et al., 2001

Rifampicin Chemiluminescence 3.2 ng/mL Halvatzis S.A et al., 1993

Rifampicin Electrochemistry 5 ng/mL Lomillo M.A.A et al., 2001

Rifampicin HPLC 0.16 µg/mL Swart K.J et al., 1992

Rifampicin HPLC 1.9 µg/mL Panchagnula R et al., 1999

Rifampicin HPLC 4.9 µg/mL Calleja I et al., 2004

Rifampicin HPLC 0.2 µg/mL Riva E et al., 1992

Rifampicin HPLC 3 µg/mL Calleri E et al., 2002

Rifampicin Fluorescence 0.2 µg/mL This study

Table S3:

| **Concentration** | **Test-paper1** | **Test-paper2** | **Test-paper3** | **Average** |
| --- | --- | --- | --- | --- |
| 0.5 µg/mL | 90.9%±0.6 | 91.8%±1.1 | 90.8±3.3 | 91%±1 |
| 5 µg/mL | 83%±2.8 | 80.7%±0.4 | 81.6±10.7 | 82%±1.1 |
| 10 µg/mL | 83.3%±1.5 | 80%±1.6 | 80.6±0.6 | 81%±1.8 |
| 30 µg/mL | 82.2%±5.4 | 75.5%±1.6 | 79.9±0.9 | 79%±3.4 |
| 50 µg/mL | 78.7%±3.1 | 75.8%±3 | 78.9±0.6 | 78%±1.7 |
| 100 µg/mL | 74.3%±4 | 75.7%±0.6 | 76±2.2 | 75%±0.3 |
| 500 µg/mL | 70.8%±4.6 | 74.7%±0.4 | 73.5±3.5 | 73%±1.9 |
| 1000 µg/mL | 68%±2.9 | 70%±0.5 | 69.6±3.1 | 69%±1.1 |
